# Dried plum mitigates spinal cord injury‐induced bone loss in mice

**DOI:** 10.1002/jsp2.1113

**Published:** 2020-07-15

**Authors:** Xuhui Liu, Mengyao Liu, Russell Turner, Urszula Iwaniec, Hubert Kim, Bernard Halloran

**Affiliations:** ^1^ San Francisco Veterans Affairs Medical Center Department of Veterans Affairs San Francisco California USA; ^2^ Department of Orthopedic Surgery University of California at San Francisco San Francisco California USA; ^3^ Skeletal Biology Laboratory, College of Public Health and Human Science Oregon State University Corvallis Oregon USA; ^4^ Department of Medicine University of California at San Francisco San Francisco California USA

**Keywords:** bone loss, dried plum, microCT, spinal cord injury

## Abstract

Spinal cord injury (SCI) is accompanied by rapid loss of bone and increased risk of low impact fractures. Current pharmacological treatment approaches have proven to be relatively ineffective in preventing or treating bone loss after SCI. Dietary supplementation with dried plum (DP) has been shown to have dramatic effects on bone in various other disease models. In this study, we tested the efficacy of DP in preventing bone loss after SCI and restoring bone that has already been lost in response to SCI. Male C57BL/6J mice (3‐month‐old) underwent SCI and were fed a diet containing 25% DP by weight or a control diet for up to 4 weeks to assess whether DP can prevent bone loss. To determine whether DP could restore bone already lost due to SCI, mice were put on a control diet for 2 weeks (to allow bone loss) and then shifted to a DP supplemented diet for an additional 2 weeks. The skeletal responses to SCI and dietary supplementation with DP were assessed using microCT analysis, bone histomorphometry and strength testing. Dietary supplementation with DP completely prevented the loss of bone and bone strength induced by SCI in acutely injured mice. DP also could restore a fraction of the bone lost and attenuate the loss of bone strength after SCI. These results suggest that dietary supplementation with DP or factors derived from DP may prove to be an effective treatment for the loss of bone in patients with SCI.

## Graphical abstract

Spinal cord injury (SCI) is accompanied by rapid loss of bone and increased risk of low impact fractures. In this study, we showed that diet supplement of dried plum (DP) completely prevented bone loss and restored a fraction of the bone lost after SCI in a mouse model. These results suggest that dietary supplementation with DP or factors derived from DP may be an effective treatment for the loss of bone after SCI.

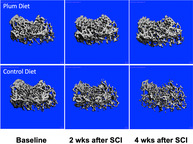

## 1 INTRODUCTION

Patients with spinal cord injuries (SCI) rapidly lose bone.^1^, ^2^ Bone formation rates can decrease as fast as 1% per week and sublesional bone mineral density (BMD) can decrease by as much as 40% in SCI patients.^3^, ^4^ Bone loss after SCI leads to increased risk of low impact fractures and significantly increases the morbidity and mortality of SCI patients.^5^ It has been reported that as high as 52% of SCI patients are diagnosed as osteoporotic within 12 months of injury.^6^ Fractures most often involve the tibia or fibula, but upper extremity fractures also occur, most commonly in those with higher cord lesions. Falls from a wheelchair and transfers are the most common causes of fracture, although fractures can also result from low‐impact activities, such as performing range‐of‐motion activities.^3^ Fracture healing is more challenging for SCI patients compared to healthy patients. It is reported that as high as 83% of long bone fractures in SCI patients require operative management.^7^

Pharmacological treatments for the loss of bone in SCI patients have been relatively ineffective. Vitamin D supplementation to restore vitamin D levels in individuals with vitamin D deficiency is commonly used but has not been effective in preventing and restoring the bone loss associated with SCI.^8^ Despite some success, the effects of the bisphosphonates have been inconsistent. A recent meta‐analysis containing 19 studies involving 364 patients showed that acute SCI participants treated with bisphosphonate therapy demonstrated a trend toward less bone loss.^9^ Bauman et al. found that zoledronic acid did not prevent BMD loss at the knee in SCI patients.^10^ Denosumab, a human monoclonal antibody to RANKL, has been shown to decrease bone turnover and a recent study showed that Denosumab decreases bone turnover markers in patients with recent SCI.^11^ However, its long‐term effect in preventing bone loss and restoring bone after SCI remain unknown. Teriparatide (recombinant PTH 1‐34) has also been studied but the effects in patients with SCI are unclear. Teriparatide combined with robotically assisted gait training was evaluated in 12 chronically injured nonambulatory subjects with low bone mass. Trabecular thickness increased at the knee at 3 months but not 6 or 12 months. BMD measurements after treatment at the spine and hips were not statistically significant.^12^ In a recent larger clinical trial in patients with chronic SCI, Teriparatide treatment resulted in a significant increase in spine BMD at 1 year and improvements to the hip at 2 years.^13^

Although some of these studies are promising, issues with patient compliance, adverse side effects, cost and long‐term efficacy remain. The use of specific foods and nutritional supplements has gained increasing attention as alternate treatment approaches. We have shown that dietary supplementation with dried plum (DP) can have dramatic effects on bone.^14^, ^15^, ^16^, ^17^, ^18^ Dried plum diets can increase cancellous bone volume in adult and aged mice by 65% and 33%, respectively. Dried plum also improves bone strength and prevents bone loss and restores bone already lost due to estrogen deficiency in rats.^19^ Although limited, studies in humans also suggest that dietary DP can increase BMD.^20^

To determine whether dietary supplementation with DP can prevent the loss of bone induced by SCI, and restore bone that has already been lost in response to SCI, we fed mice a diet containing 25% DP in a mouse model of SCI.

## 2 MATERIALS AND METHODS

### 2.1 Animals

Male C57BL/6J mice (3 months of age) were obtained from the Jackson Laboratory (Sacramento, CA). The animals were housed in air‐filtered, humidity‐ and temperature‐controlled rooms with equal 12 hours light‐12 hours dark cycles and fed a standard mouse diet before the study began. All mice went through a one‐week acclimatization period after arriving at our facility before the experiments were conducted. In total, 53 mice were used in this study (including 45 with SCI and 8 without SCI). The detailed animal groups and numbers are listed in Table 1. The animal protocol for the study was in accordance with the NIH Guide for the Care and Use of Laboratory Animals and approved by the Animal Care and Use Committee at the Veterans Affairs Medical Center, San Francisco.

**TABLE 1:** Animal groups and numbers in this study

Experiment	Group	Diet	SCI	Number of mice (N)
Prevention	1	Control	Yes	N = 9 (2 were removed due to incomplete SCI)
	2	Plum	Yes	N = 9 (1 was removed due to incomplete SCI)
Treatment	1	Control	No	N = 8
	2	Control (2 weeks)	Yes	N = 9 (1 was removed due to incomplete SCI)
	3	Control (4 weeks)	Yes	N = 9 (1 was removed due to incomplete SCI)
	4	2 weeks control diet, then 2 weeks of plum diet	Yes	N = 9 (1 was removed due to incomplete SCI)
Total				N = 53

### 2.2 Spinal Cord Injury

Mice underwent a dorsal midthoracic laminectomy followed with spinal cord contusion using a modified Allen weight‐drop method as described previously.^21^ The injury was induced by dropping a 35 g stainless steel rod onto the exposed spinal cord at the T10 level with a penetrating depth of 1.8 mm from a height of 50 mm, generating a complete paraplegia. Animals were under anesthesia with 1‐4% isoflurane in oxygen inhalation during all procedures. In total, 45 mice underwent spinal cord contusion procedure. Six of them were excluded from the study due to incomplete SCI (attrition rate = 14.3%). Thirty nine of the mice with complete SCI were used in this study. Bupivacaine (0.25%, 0.1 mL) was injected to the incision site before surgery and Buprenorphine (0.1 mg/kg) was injected twice a day after surgery for the first two postoperational days.

### 2.3 Plum Diet

DP was received as a kind gift from the California Dried Plum Board. Mice were fed either a control diet (AIN‐93 M) or a diet containing 25% DP by diet weight (AIN‐93 M control diet containing 25% DP, w/w) for 2 or 4 weeks. The DP diet was selected based on its effectiveness to alter bone turnover in previous studies.^14^, ^16^ Control and experimental diets were formulated in a pellet form and contained an equal amount of energy, protein, fat, carbohydrate, calcium, phosphorus and other nutrients. Details of the diets are published elsewhere.^14^ The mice have access to the food at all time in the cage.

### 2.4 Animal protocols

Our objectives were to determine: 1) whether dietary supplementation with DP can prevent the loss of bone induced by SCI (prevention experiment) and 2) whether the bone lost after SCI can be replaced by switching to a DP diet (recovery experiment). To accomplish our first objective mice were divided into two groups (control diet and DP diet, N = 7 in control diet group and N = 8 in DP diet group) and fed their respective diets beginning immediately after SCI. in vivo microCT scanning was performed three times, at base line (immediately before SCI) and 2 and 4 weeks after SCI. Body weight was measured at the time of scanning. To accomplish our second objective we divided mice into 4 groups: group 1 was euthanized at baseline without SCI, group 2 underwent SCI and was fed the control diet for 2 weeks, group 3 underwent SCI and was fed the control diet for 4 weeks and group 4 underwent SCI and was fed the control diet for 2 weeks (to allow bone loss) and then switched to the DP diet for an additional 2 weeks. To test our hypothesis, we compared the mice switched to the DP diet (group 4) to the control diet groups at 2 weeks (group 2) and 4 weeks (group 3). Comparing group 2 to group 4 allowed us to determine whether we could restore bone that had been lost. Comparing group 3 to 4 allowed us to determine whether DP can improve bone volume and strength. There were 8 mice in each group. Mice were injected subcutaneously with Calcein (10 mg/kg) and Demeclocycline (10 mg/kg) (both from Sigma‐Aldrich, St Louis, MO, USA) 7 and 2 days before euthanasia, respectively, to label bone mineralizing surfaces. At the time of euthanasia body weights were recorded and the left femurs were fixed in Zinc‐buffered 10% neutralized formalin (Z‐fix) (ThermoScientific, Waltham, Massachusetts) for 24 hours and dehydrated with 70% ethanol followed by microCT scanning and histomorphometry. The right femurs were wrapped with PBS‐soaked gauze and frozen at −20°C in a sealed container for mechanical testing later.

### 2.5 MicroCT analysis

MicroCT analysis was conducted using a Scanco Viva CT 40 (Scanco Medical, Basserdorf, Switzerland) microCT. in vivo scanning was conducted with the isotropic voxel size of 10.5 μm and the x‐ray energy of 55 kVp. ex vivo scanning was conducted with 70 kVp and 85 μA. The isotropic resolution was 10.5 μm. The region of interest (ROI) for the distal femur consisted of the cancellous bone compartment beginning 0.5 mm proximal to the growth plate and extending proximally 1.5 mm. A threshold was determined as 22% of the maximal gray scale to distinguish mineralized from soft tissue. Trabecular bone volume expressed as a percent of total volume (BV/TV, %), trabecular number (Tb.N, 1/mm), trabecular thickness (Tb.Th, μm), structure model index (SMI; ranges from 0‐3, with 0 = plate‐like and 3 = rod‐like) and connectivity density (Conn.D, 1/mm^3^) were evaluated using the software provided by the manufacturer. The diaphysis of femurs from animals in the prevention experiment was also scanned and the thickness of cortical bone was measured at the end of the experiment.

### 2.6 Mechanical Testing

After the frozen bones were thawed, three‐point bending was used to assess bone strength in the femoral diaphysis using a Bose ElectroForce 3220 (Bose, Corp. Framingham, Massachusetts). The methods have been described previously.^22^ The span between the left overhang and right overhang was 7.65 mm. The Maximum Load (N) and stiffness (N/mm) were recorded using the manufacturers' software.

### 2.7 Bone Histomorphometry

The distal end of the left femur was processed undecalcified for quantitative bone histomorphometry as described previously.^23^ Bones were fixed in Z‐fix for 24 hours, dehydrated in increasing concentrations of ethanol and embedded undecalcified in modified methyl methacrylate. Coronal sections (4 μm thick) were cut with a vertical bed microtome (Leica 2065) and affixed to gel coated slides. Osteoblast (Ob.Pm/B.Pm, %) and osteoclast (Oc.Pm/B.Pm, %) perimeters were measured in sections stained for TRAP and counter stained with toluidine blue. Fluorochrome‐based indices of bone formation including mineralizing perimeter (M.Pm/B.Pm, %), mineral apposition rate (MAR, μm/d) and perimeter‐based bone formation rate (BFR/B.Pm, dL.Pm + ½ sL.Pm/B.Pm, μm^2^/μm/d) were measured in unstained sections. Histomorphometric data were collected using the OsteoMeasure System (OsteoMetrics, Inc., Atlanta, Georgia). The ROI included cancellous bone beginning 0.25 mm proximal to the growth plate and extending proximally 1.0 mm. Two‐dimensional histomorphometric terminology is in accordance with recommendations by the ASBMR Histomorphometry Nomenclature Committee.^24^ One section per sample was quantified.

### 2.8 Statistical analysis

Data are reported as mean ± SD with n = 7‐8 mice per experimental group. A two‐way ANOVA and a Holm‐Sidak post hoc test were used in the prevention studies to compare groups. A Dunnet's test was used in the recovery studies to compare the switched diet group to the control diet groups at 2 and 4 weeks. Normality (Shapiro‐Wilk) and equal variance were assessed for all groups. Statistical significance was considered when *P* < .05.

## 3 RESULTS

**FIGURE 1:**
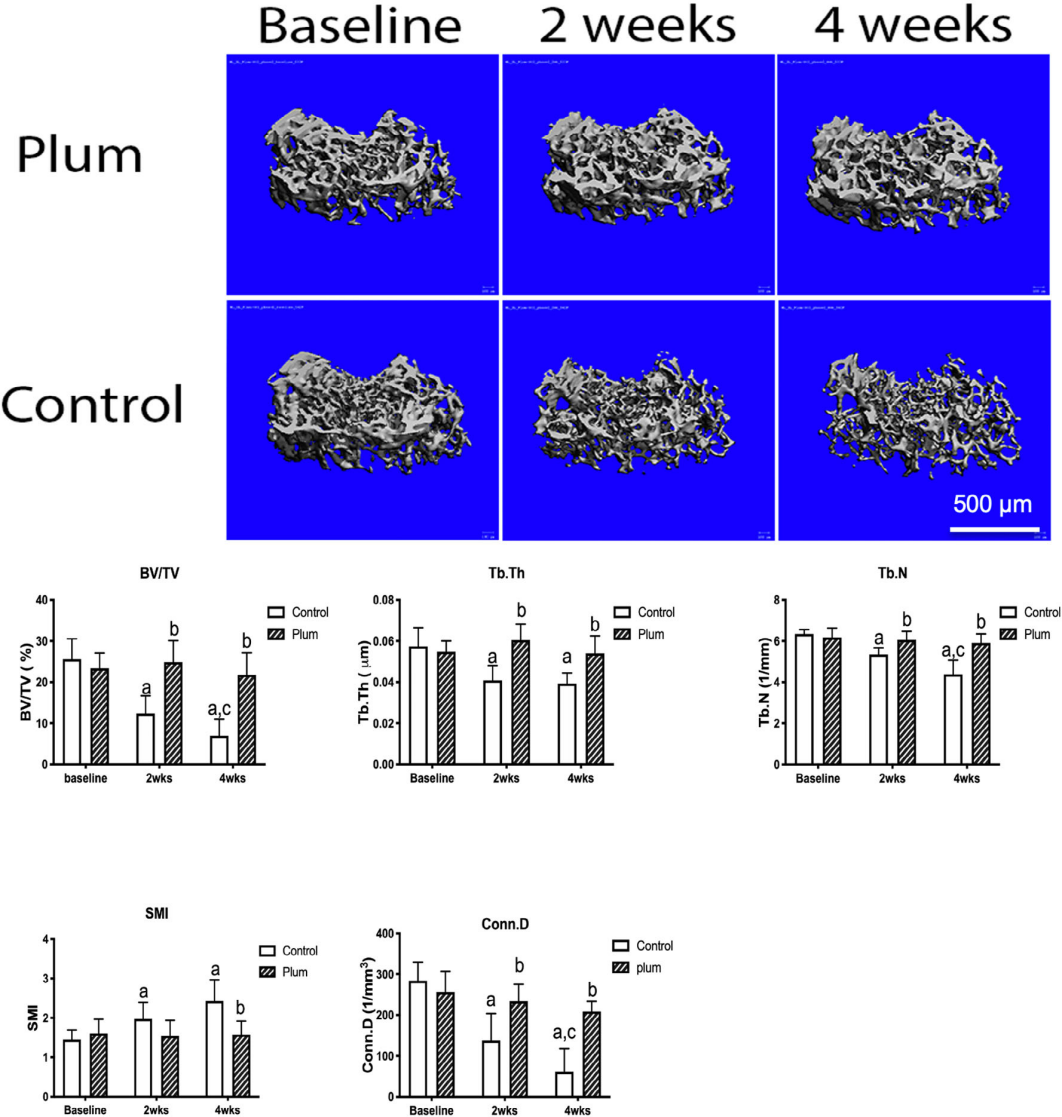
Top: Typical uCT images of the distal femoral metaphysis at baseline, and in mice fed a control diet or a diet containing 25% DP for 2 or 4 weeks after SCI. Mice fed the control diet showed a progressive loss of bone after SCI. Mice fed the DP diet showed no loss of bone. Bottom: Quantitation of uCT results. Cancellous bone volume (BV/TV) and micro‐architecture (Tb.Th, Tb.N, Conn.D, SMI) from μCT analysis in the distal femoral metaphysis in mice fed a control diet or a diet containing 25% DP for 2 or 4 weeks (mean + SD, n = 7‐8). Data were analyzed using 2‐way ANOVA. P‐values for time, dose of DP and interaction were significant (*P* < .05) for all variables. ^a^ *P <* .05 compared to baseline and, ^b^ *P <* .05 compared to control diet at the same time point and ^c^ *p* < .05 compared to the control diet at 2 weeks). DP protected against bone loss and changes in the microarchitecture induced by SCI

**FIGURE 2:**
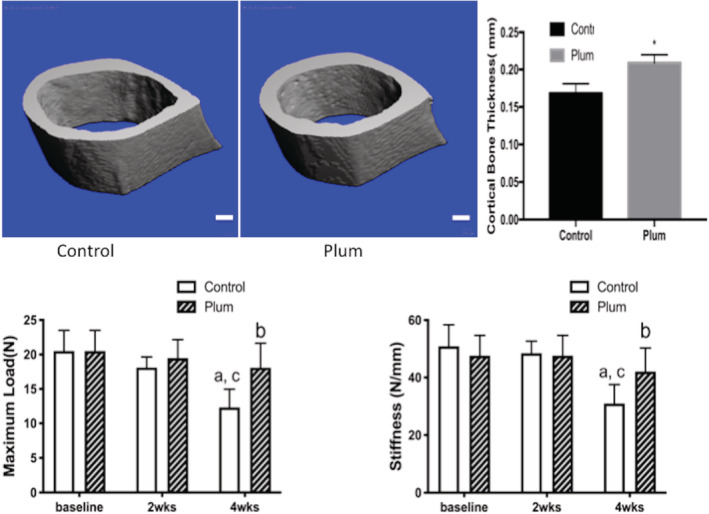
Top: MicroCT scanning of diaphysis of femurs showed that mice in the plum diet group have significantly thicker cortical bone at 4 weeks after SCI compared to those in the control diet group (*, *P* < .05) (scale bar: 200 μm). Bottom: Maximum load and stiffness of the mid‐femoral diaphysis measured with three‐point bending in mice fed a control diet or a diet containing 25% DP for 2 or 4 weeks (mean + SD, n = 7‐8). Data were analyzed using 2‐way ANOVA. P‐values for time, dose of DP and interaction were significant (*P* < .05). ^a^ *P* < .05 compared to baseline and, ^b^ *P <* .05 compared to control diet at the same time point and ^c^ *P* < .05 compared to the control diet at 2 weeks). DP protected against loss of bone strength induced by SCI

**FIGURE 3:**
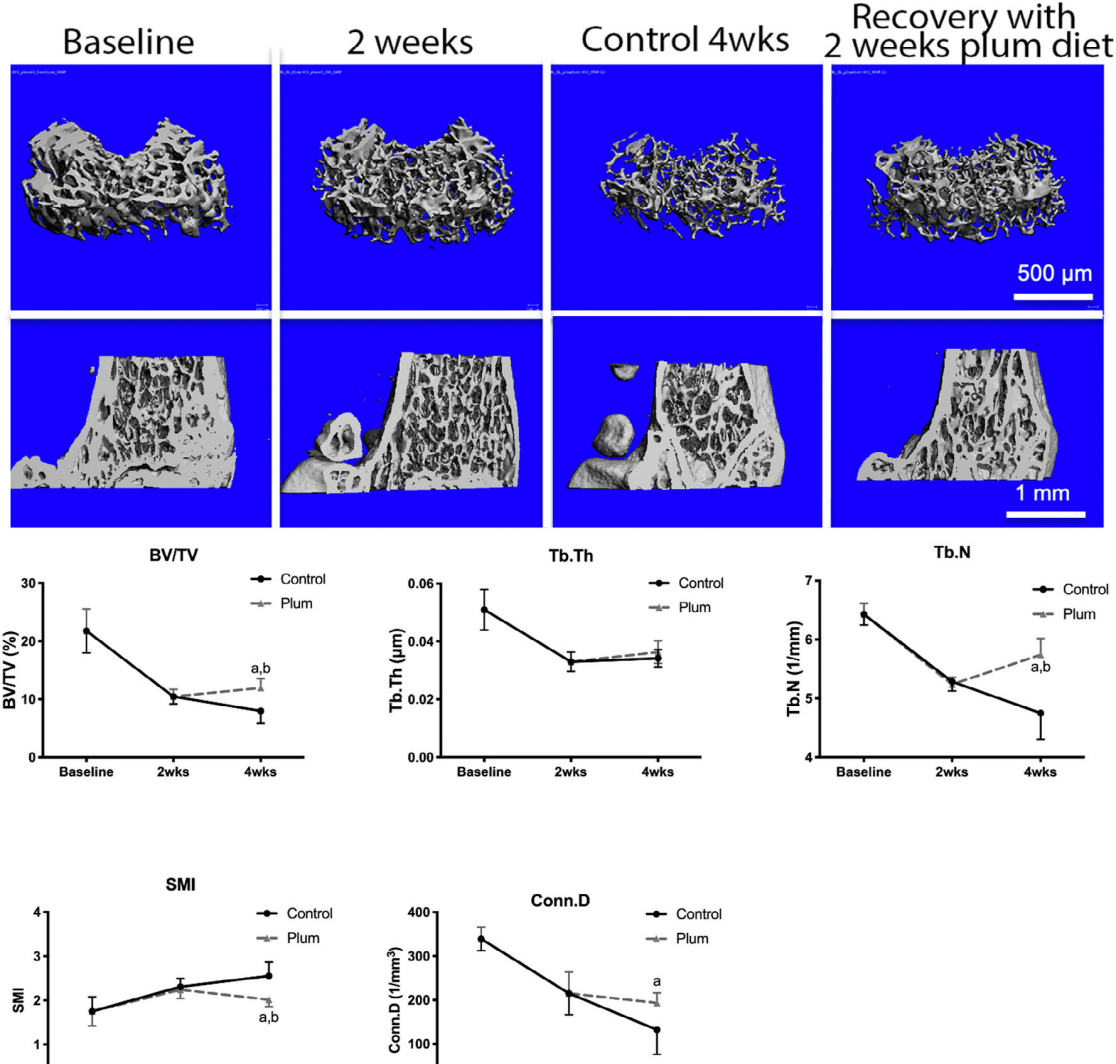
Top: Typical uCT images (cross section views in the upper row and sagittal section views in the lower row) of the distal femoral metaphysis at baseline and in mice fed a control diet or diet containing 25% DP for 2 or 4 weeks after SCI, and a group of mice fed the control diet for 2 weeks and then switched to the DP diet for 2 more weeks. DP restored a fraction of the bone lost and attenuated the changes in microarchitecture induced by SCI. Bottom: Quantification of diaphysis of femurs results. Cancellous bone volume (BV/TV) and micro‐architecture (Tb.Th, Tb.N, Conn.D, SMI) from μCT analysis in the distal femoral metaphysis in mice fed the control diet for 2 or 4 weeks and mice fed the control diet for 2 weeks and then switched to the DP diet for 2 more weeks (mean with error bar for SD. n = 8 in each group, ^a^ *p <* .05 compared to mice on the control diet for 4 weeks and, ^b^ *P <* .05 compared to mice on the control diet for 2 weeks)

Dietary supplementation with DP completely prevented the loss of bone and bone strength induced by SCI (Figures 1, 2, 3). MicroCT images at baseline, 2 and 4 weeks after SCI in mice fed either the DP diet or the control diet are shown in Figure 1. Mice fed the control diet lost 53% of their cancellous bone volume by 2 weeks after SCI. Four weeks after SCI, the loss was 71%. No change in cancellous bone volume compared to baseline was detected in the mice fed the DP diet at either 2 or 4 weeks after SCI. Trabecular thickness and number and connectivity density decreased in the control diet mice but not in the DP diet mice. The SMI increased in the control mice but did not change in the DP fed mice. Bone strength was significantly reduced in the control diet mice after 4 weeks but not after 2 weeks. No changes in strength in the plum diet mice were detected. Compared to those in the control diet group, mice in the plum diet group have significantly higher thickness of cortical bone at 4 weeks after SCI (Figure 2).

**FIGURE 4:**
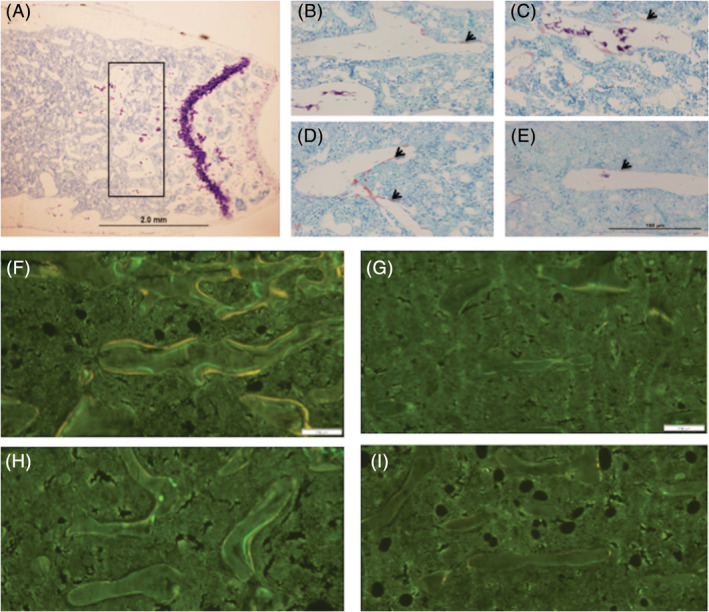
Typical histomorphology images. A, A low magnification overview of the femur indicating the region of interest. B‐E, TRAP staining images (counter stained with toluidine blue) and, F‐I, fluorochrome images of the distal femoral metaphysis at baseline (B, F) and in mice fed a control diet for 2 (C, G) or 4 weeks after SCI (D, H), and a group of mice fed the control diet for 2 weeks and then switched to the DP diet for 2 more weeks (E, I). Osteoclast surface was indicated with arrows

**FIGURE 5:**
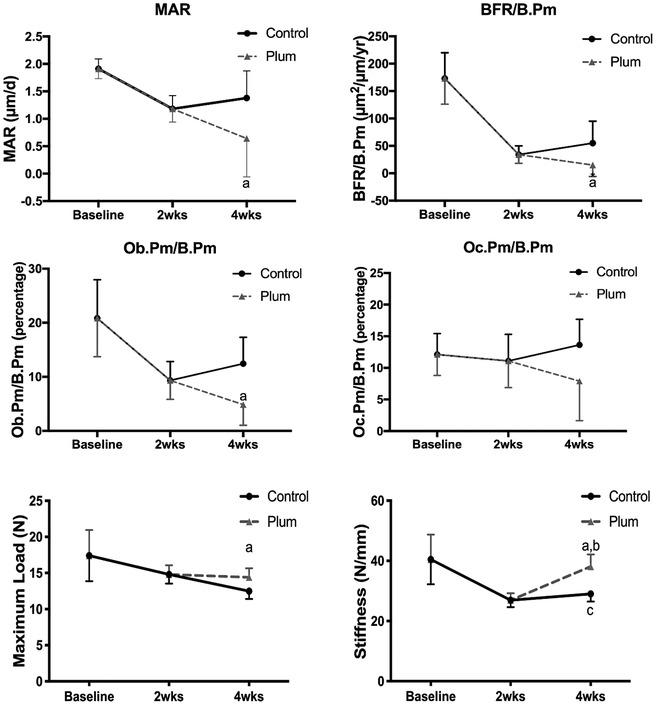
Bone histomorphometry (MAR, mineral apposition rate; BFR/B.Pm, bone formation rate/bone perimeter; Ob.Pm/B.Pm, osteoblast perimeter/bone perimeter; Oc.Pm/B.Pm, osteoclast perimeter/bone perimeter) in the distal femoral metaphysis and the maximum load and stiffness of the mid‐femoral diaphysis measured with three‐point bending in mice of baseline, fed a control diet for 2 or 4 weeks, and mice fed with control diet for 2 weeks and then switched to the DP diet for 2 more weeks after SCI (mean with error bar for SD, n = 8 in each group, ^a^ *P <* .05 compared to mice on the control diet for 4 weeks)

To determine whether DP could restore bone after it had already been lost from SCI, we compared mice after 2 and 4 weeks on the control diet to mice fed the control diet for 2 weeks and then switched to the DP diet for 2 more weeks. MicroCT images and quantitative analyses are shown in Figure 3. Switching to the DP diet at 2 weeks after SCI restored a fraction of the bone lost. Trabecular number, but not thickness was also restored in part. Switching to the DP diet also attenuated the changes in SMI, tissue density and connectivity density. TRAP staining and quantitative analyses of bone histomorphology are shown in Figures 4 and 5. The MAR, BFR and osteoblast perimeter were lower in the mice switched to the DP diet when compared to the mice fed the control diet for 4 weeks. There were no significant changes in osteoclast perimeter. Bone strength was greater in mice switched to the DP diet when compared to mice fed the control diet for 4 weeks (Figure 5).

In the prevention experiment, all mice lost weight following SCI. The body weights of mice in the control diet group were 26.3 ± 1.5 g (baseline), 23.0 ± 1.9 g (2 weeks after SCI) and 21.5 ± 2.5 g (4 weeks after SCI). The body weights of mice in the plum diet group were 26.1 ± 1.7 g (baseline), 22.2 ± 1.8 g (2 weeks after SCI) and 20.8 ± 1.6 g (4 weeks after SCI). Two‐way ANOVA analysis showed a significant effect of time (*P* < .01), but not diet. In the recovery experiment, the body weights of mice in each group were: 24.9 ± 1.2 g (baseline), 19.9 ± 1.2 g (2 weeks control diet), 22.2 ± 1.2 g (4 weeks control diet) and 22.9 ± 1.7 g (2 weeks control diet +2 weeks plum diet). Dunnet's test showed a significant difference in body weights (*P* < .01) between mice fed the control diet for 2 weeks and mice fed the control diet for 2 weeks and then switched to the DP diet. There was no difference in body weights between mice fed the control diet for 4 weeks and mice in the switched diet group.

## 4 DISCUSSION

The results of our studies suggest that dietary supplementation with DP can completely prevent the loss of bone induced by SCI in a mouse model. The results show that the loss of bone following acute SCI is associated with trabecular thinning and a lower trabecular number. In DP fed mice, trabecular thickness and number were preserved. These findings are similar to those observed in normal uninjured mice.^14^ The loss of bone in the control diet mice was associated with decreases in the mineral apposition and bone formation rates, osteoblast perimeter and a small but not significant increase in the osteoclast perimeter. The loss of bone appears to be primarily a consequence of decreased osteoblastic activity and number, although an increase in osteoclastic activity cannot be excluded. Feeding DP further decreased the mineral apposition and bone formation rates and the osteoblast perimeter suggesting that the preventative effects of DP diet are not likely due to increased bone forming activity. In a previous study using uninjured mice, dietary supplementation with DP decreased osteoclast perimeter and serum concentration of type 1 collagen C‐terminal telopeptide (CTX) (a marker of bone resorption).^14^ These data suggest that preservation of bone in our SCI mouse model may be due in part to suppression of osteoclastic activity. In the mice receiving the control diet the SMI increased following SCI, suggesting that the cancellous bone in the femur is becoming more rod‐like as bone is lost. Dietary DP prevented this and promoted a more normal cancellous bone structure. Bone strength decreased following SCI in mice consuming a normal diet, a finding consistent with the decrease in bone volume. This was mitigated in mice receiving the DP diet. The preservation of strength is consistent with the maintenance of bone mass.

Preservation of bone mass and strength is critical in the acutely injured patient. In chronically injured patients who have already lost bone the goal is to prevent further loss or restore the bone lost. Dietary supplementation with DP beginning 2 weeks after SCI and after a deficit of bone had been established (−40%), restored a fraction of the lost bone. This was associated with an increase in trabecular number but not trabecular thickness suggesting that the DP diet is increasing trabecular bridging but not adding new bone to trabecular surfaces. Importantly a DP diet can prevent bone loss when treatment is started after a bone deficit has occurred and may even be able to restore some lost bone but complete restoration is unlikely. Longer DP treatment periods, however, may promote further bone recovery.

Although DP supplemented diets can protect against bone lost following SCI, the mechanisms involved are poorly understood. Our previous findings suggest that in mature mice the primary cellular effector for the increase in bone volume is a decrease in osteoclastic number and bone resorption.^13^ Consistent with this idea, DP has been shown to alter allocation of cells in the hematopoietic lineage and inhibit recruitment of osteoclast precursors into the osteoclast lineage.^24^ It appears that the gain in bone occurs because of bone formation and resorption where the decrease in resorption is greater than the decrease in formation.

All mice in the prevention experiment lost weight, a finding consistent with previous experiments, but there was no difference in the amount of weight loss between control and DP fed mice. These observations suggest that the preservation of bone by DP supplementation was not a consequence of preservation of weight. Furthermore, body weights in the recovery experiment were not different between control diet mice at 4 weeks and mice switched to the DP diet. These results suggest that the fractional recovery of bone was not a consequence of an increase in body weight.

The bioactive factors in DP have not been identified. Previous studies suggest that the effects of DP on bone may be mediated through DP‐specific polyphenols, although other nonphenolic compounds are likely to also be involved.^18^, ^25^ In DP fed mice the serum level of several polyphenols, including quercetin, increases and quercetin has been shown to decrease bone loss in both diabetic rats^26^ and ovariectomized mice.^27^ Curcumin, another polyphenol has been reported in both preclinical^28^ and clinical^29^ studies to inhibit bone loss in patients following SCI. Though DP does not contain a high concentration of calcium and almost no Vitamin D, it does contain a relative high concentration of vitamin K, an important factor in bone health.^30^ Future work is warranted to identify the bioactive factors in plum that improve bone health.

SCI patients frequently display neurogenic bowel dysfunction due to the loss of central nervous system control to the GI system. Gungor et al. showed that Pseudobutyrivibrio and Megamonas genera were significantly lower in SCI patients with bowel dysfunction compared to healthy groups, suggesting an alteration of the gut microbiome after SCI.^31^ Disruption of the microbiome, a process known as dysbiosis, after SCI has been considered as a disease‐modifying factor^32^ and fecal transplant prevented gut dysbiosis and anxiety‐like behavior after spinal cord injury in rats.^33^ However, the role of dysbiosis in SCI‐induced osteopenia remains unclear.

Dried plum contains a complex of prebiotic factors and changes in the microbiome have been linked to changes in mineral metabolism.^34^, ^35^, ^36^, ^37^ Intestinal calcium bioavailability has also been shown to be associated with changes in short chain fatty acids (SCFA),^36^ although dietary consumption of plum has been reported to have no effect on SCFA.^38^ Other factors reported to affect bone, such as vitamin K (menaquinone), are produced in the intestine and synthesis is altered by the composition of the intestinal microbiota.^39^, ^40^ Collectively, these observations suggest that the effects of DP on bone in SCI patients may be mediated through changes in the gut microbiome.

In conclusion, dietary supplementation with DP can prevent bone loss in an acute model of SCI, and restore a fraction, but not all, of the bone that has already been lost in mice following SCI. A clinical trial treating SCI patients with DP is currently underway. If shown effective, dietary supplementation with DP or drugs derived from DP may prove to be an effective treatment for the loss of bone induced by SCI.

## Supporting information


**Appendix** **S1**: Supporting information
